# The *Escherichia coli* Fur pan-regulon has few conserved but many unique regulatory targets

**DOI:** 10.1093/nar/gkad253

**Published:** 2023-04-07

**Authors:** Ye Gao, Ina Bang, Yara Seif, Donghyuk Kim, Bernhard O Palsson

**Affiliations:** Department of Biological Sciences, University of California San Diego, La Jolla, CA 92093, USA; Department of Bioengineering, University of California San Diego, La Jolla, CA 92093, USA; School of Energy and Chemical Engineering, Ulsan National Institute of Science and Technology (UNIST), Ulsan 44919, Republic of Korea; Department of Bioengineering, University of California San Diego, La Jolla, CA 92093, USA; School of Energy and Chemical Engineering, Ulsan National Institute of Science and Technology (UNIST), Ulsan 44919, Republic of Korea; Schools of Life Sciences, Ulsan National Institute of Science and Technology (UNIST), Ulsan 44919, Republic of Korea; Department of Bioengineering, University of California San Diego, La Jolla, CA 92093, USA; Department of Pediatrics, University of California San Diego, La Jolla, CA 92093, USA; Novo Nordisk Foundation Center for Biosustainability, Technical University of Denmark, Building 220, Kemitorvet, 2800, Kongens Lyngby, Denmark

## Abstract

While global transcription factors (TFs) have been studied extensively in *Escherichia coli* model strains, conservation and diversity in TF regulation between strains is still unknown. Here we use a combination of ChIP-exo–to define ferric uptake regulator (Fur) binding sites–and differential gene expression–to define the Fur regulon in nine *E. coli* strains. We then define a pan-regulon consisting of 469 target genes that includes all Fur target genes in all nine strains. The pan-regulon is then divided into the core regulon (target genes found in all the strains, n = 36), the accessory regulon (target found in two to eight strains, n = 158) and the unique regulon (target genes found in one strain, n = 275). Thus, there is a small set of Fur regulated genes common to all nine strains, but a large number of regulatory targets unique to a particular strain. Many of the unique regulatory targets are genes unique to that strain. This first-established pan-regulon reveals a common core of conserved regulatory targets and significant diversity in transcriptional regulation amongst *E. coli* strains, reflecting diverse niche specification and strain history.

## Graphical Abstract

**Graphical Abstract:**
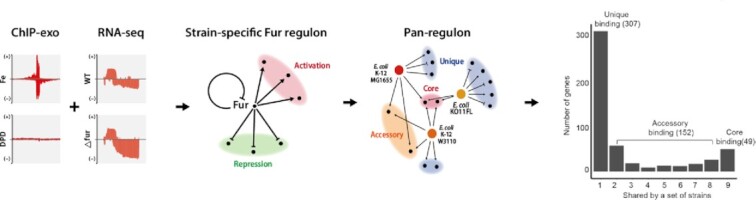
The set of commonly regulatory target genes of Fur is consistent and related to the regulation of iron metabolism. Meanwhile, the high number of uniquely regulatory targets of Fur showed diversity in Fur regulation among the nine *E. coli* strains. This diversity likely results in physiological differences between strains.

## INTRODUCTION

Transcription factors (TFs) regulate gene expression by direct or indirect activation or repression of their regulatory target genes. Many global TFs are highly conserved, and they play defining physiological roles in bacteria, enabling them to adapt to changing environments (1). Iron is a critical cofactor for many enzymes and is found in other key proteins in bacteria (2), making iron homeostasis a key regulatory priority. Ferric uptake regulator (Fur) is thus a major TF in bacteria that maintains iron homeostasis. To achieve its regulatory function, Fur coordinates many cellular processes, including oxidative stress response, anaerobic metabolism, and virulence (3).

Previous studies have investigated genome-wide binding sites of Fur in *Escherichia coli* K-12 strains (4). However, the degree of conservation of Fur transcriptional regulation between *E. coli* strains is unknown. There is no experimental evidence available that shows if the genome-wide binding patterns of Fur are maintained or are divergent within strains or phylogroups of the *E. coli* species. These regulatory relationships are often complex, rendering the results from a variety of computational predictions unreliable (5). A detailed multi-strain experimental assessment of Fur binding sites is thus needed to determine its strain-specific and species-wide regulatory functions.

The pangenome of a species is composed of the ‘core’ genome (genes conserved in all strains), ‘accessory’ genome (genes shared by more than one strains but not all the strains), and ‘unique’ genome (genes only present in one strain). As an analogy, we defined the ‘pan-Fur regulon’ as the totality of genes controlled by Fur across a set of strains studied. Similarly, the pan-regulon is composed of the core, accessory, and unique regulons. The size and the functionalities of the *E. coli* pan-regulon for any TF remain unknown, as high quality experimental *in vivo* data is not available.

Here, we perform a pan-regulon analysis of Fur combining genome-wide binding site profiling (using ChIP-exo) and differential gene expression (RNA-seq between wild-type and Fur deletion strains) to reconstruct the strain-specific Fur regulons for nine *E. coli* strains. They cover five phylogenetic groups (A, B1, B2, D and E) and include industrial (non-pathogenic) and pathogenic strains in *E. coli*. We then compile a pan-regulon from these nine strain-specific Fur regulons. This pan-regulon allows us to compare Fur regulation across different strains to determine conservation of Fur regulation within the species and to define strain-specific regulatory effects.

## MATERIALS AND METHODS

### Bacterial strains, media and growth conditions

All strains (wild type, myc-tagged, and knockout strains) used in this study are listed in Dataset 1. For ChIP-exo experiments, the strains harboring 8-myc were generated by a λ red-mediated site-specific recombination system targeting the C-terminal region as described previously (6). For expression profiling by RNA-seq, *fur* deletion strains from all strains were also constructed by a λ red-mediated site-specific recombination system (7).

For normal conditions, a M9 minimal medium (47.8 mM Na_2_HPO_4_, 22 mM KH_2_PO_4_, 8.6 mM NaCl, 18.7 mM NH_4_Cl, 2 mM MgSO_4_ and 0.1 mM CaCl_2_) with 0.2% (w/v) glucose was supplemented with 1 mL trace element solution (100X) containing 1 g EDTA, 29 mg ZnSO_4_.7H_2_O, 198 mg MnCl_2_.4H_2_O, 254 mg CoCl_2_.6H_2_O, 13.4 mg CuCl_2_ and 147 mg CaCl_2_. The culture was incubated at 37°C overnight with agitation and then was used to inoculate the fresh medium (1/200 dilution) in a 500 mL shaker flask. The volume of the fresh medium was 100 ml for each biological replicate. The fresh culture was incubated at 37°C with the agitation at 300 rpm to the mid-log phase (OD_600_ ≈ 0.5), and then collected for the experiments.

For ChIP-exo experiments, glycerol stocks of 8-myc strains were inoculated into an M9 minimal medium with 0.2% (w/v) glucose. The volume of the fresh medium was 100 mL for each biological replicate. In this study, there are two conditions for ChIP-exo experiments. One is the iron-replete condition: M9 minimal medium was supplemented with 0.1 mM FeCl_2_. The other is the iron-restricted condition: M9 minimal medium was supplemented with 0.2 mM 2,2’-dipyridyl at early-log phase and incubated at 37°C for two additional h with vigorous agitation. Then the cell cultures were collected for ChIP-exo experiments.

For RNA-seq expression profiling, glycerol stocks of all strains were inoculated into M9 minimal medium with the same carbon sources as used in the ChIP-exo experiment. The concentration of carbon sources was 0.2% (w/v). For iron-replete conditions, M9 minimal medium was also supplemented with 0.1 mM FeCl_2_. The culture was incubated at 37°C overnight with agitation and then was used to inoculate the fresh medium. The fresh culture was incubated at 37°C with agitation to the mid-log phase (OD_600_ ≈ 0.5), and then the culture was collected for total RNA isolation and for use in subsequent RNA-seq experiments.

### Pan-genome analysis and construction of phylogenetic tree

A total of nine complete genomic sequences of *E. coli* were downloaded from the Pathosystems Resource Integration Center database and re-annotated using Prokka (8,9). The *E. coli* pan genome was built using BPGA, an ultra-fast pipeline (10). Genes were grouped into orthologous gene families based on sequence similarity identified through the protein based Basic Local Alignment Search Tool using a cutoff of 80% percentage identity and a maximum *E*-value of 10^−6^ (11). Gene families were then functionally annotated and assigned to clusters of orthologous groups ontology using eggNOG (12). Then the orthologous groups which are conserved across all strains (core genes) but not duplicated in any of the selected strains were identified, and then visualized the phylogenetic tree in R using GGTREE (13).

### Measurement of bacterial growth

The effects of iron-limited conditions on cell growth were examined by growing all strains and Δ*fur* strains under M9 minimal glucose medium without trace element solution. Cells grown overnight on M9 minimal glucose medium at 37°C with agitation were inoculated into the fresh medium, then were incubated at 37°C with agitation. Similarly, to measure growth on iron-replete condition, the culture incubated at M9 minimal medium was supplemented with 0.1 mM FeCl_2_ at 37°C overnight with agitation and then was used to inoculate the fresh medium (1/200 dilution). The fresh culture was incubated at 37°C with agitation. All of the growth curves were measured by three independent experiments at least and recorded by OD_600_ using BioTek Microplate spectrophotometer. The growth rate was calculated by Growthcurver (14). All the data from the spectrophotometer were fitted to a standard form of the logistic equation. The significant difference between wild type and Δ*fur* strain was determined by the t test, *P* < 0.05.

### Phenotype microarrays analysis

The phenotypic fingerprints of wild type and *fur* knockout strains were recorded using the OmniLog Phenotype MicroArray (PM) platform (Biolog, Hayward, CA, USA). Each PM assay was performed on two biological replicates, in accordance with the manufacturer's instructions. Briefly, cells at the late exponential (LE) growth were collected, washed in sterile potassium phosphate buffer (50 mM, pH 7.0), and inoculated in PM11 and PM12 microplates, which account for 48 different antibiotics. Kinetic data from PM panels were automatically recorded by the OmniLog reader (Biolog) during incubation at 37°C for 48 h. Generated longitudinal data were analyzed using the Micro4Food PM pipeline (https://github.com/Neuls/omnitools).

### ChIP-exo experiments

ChIP-exo experiments were performed in biological duplicates following the procedures as below. To identify Fur binding maps for each strain *in vivo*, the DNA bound to Fur from formaldehyde cross-linked cells were isolated by chromatin immunoprecipitation (ChIP) with the specific antibodies that specifically recognize myc-tag (9E10, Santa Cruz Biotechnology), and Dynabeads Pan Mouse IgG magnetic beads (Invitrogen) followed by stringent washings as described previously (15). ChIP materials (chromatin-beads) were used to perform on-bead enzymatic reactions of the ChIP-exo method (16). The sheared DNA of chromatin-beads was repaired by the NEBNext End Repair Module (New England Biolabs) followed by the addition of a single dA overhang and ligation of the first adaptor (5’-phosphorylated) using dA-Tailing Module (New England Biolabs) and NEBNext Quick Ligation Module (New England Biolabs), respectively. Nick repair was performed by using PreCR Repair Mix (New England Biolabs). Lambda exonuclease- and RecJ_f_ exonuclease-treated chromatin was eluted from the beads and overnight incubation at 65°C reversed the protein-DNA cross-link. RNAs- and Proteins-removed DNA samples were used to perform primer extension and second adaptor ligation with following modifications. The DNA samples incubated for primer extension as described previously (17) were treated with dA-Tailing Module (New England Biolabs) and NEBNext Quick Ligation Module (New England Biolabs) for second adaptor ligation. The DNA sample purified by GeneRead Size Selection Kit (Qiagen) was enriched by polymerase chain reaction (PCR) using Phusion High-Fidelity DNA Polymerase (New England Biolabs). The amplified DNA samples were purified again by GeneRead Size Selection Kit (Qiagen) and quantified using Qubit dsDNA HS Assay Kit (Life Technologies). Quality of the DNA sample was checked by running Agilent High Sensitivity DNA Kit using Agilent 2100 Bioanalyzer (Agilent) before sequenced using HiSeq 2500 (Illumina) following the manufacturer's instructions. Each modified step was also performed following the manufacturer's instructions.

### Peak calling for ChIP-exo dataset

Each read in raw sequencing data was trimmed to 31 bp by using FASTX-toolkit (18). The sequencing reads were mapped on each reference genome using Bowtie (19). The SAM output files generated by Bowtie were changed to BAM format using SAMtools (20). The peaks were predicted by the MACE program and were directly curated by Metascope visualization (21). The calculation of S/N ratio resembles the way to calculate ChIP-chip peak intensity where IP signal was divided by Mock signal in a previous study (17). Finally, Genome-scale data were visualized using MetaScope (22) and NimbleGen's SignalMap software. Each peak was assigned to the target gene, according to genomic position. The genome-wide binding sites were listed in Dataset 2.

### Motif search from ChIP-exo peaks

The sequence motif analysis for Fur binding sites was performed using the MEME software suite (23). For each strain, sequences in binding regions were extracted from the reference genome (Dataset 1). To achieve a more accurate motif, the sequence of each binding site was extended by 10 bp at each end. The width parameter was fixed at 20 bp and the minsites parameter was fixed at 90% of the total number of the sequence. All other parameters followed the default setting.

### RNA-seq expression profiling

RNA-seq experiments were performed in biological duplicates following the procedures as below. Three milliliters of cells from mid-log phase cultures were mixed with 6 mL RNAprotect Bacteria Reagent (Qiagen). Samples were mixed immediately by vortexing for 5 s, incubated for 5 min at room temperature, and then centrifuged at 5000 *g* for 10 min. The supernatant was decanted, and any residual supernatant was removed by inverting the tube once onto a paper towel. Total RNA samples were then isolated using RNeasy Plus Mini kit (Qiagen) following the manufacturer's instructions. Samples were then quantified using a NanoDrop 1000 spectrophotometer (Thermo Scientific) and quality of the isolated RNA was checked by running RNA 6000 Pico Kit using Agilent 2100 Bioanalyzer (Agilent). Paired-end, strand-specific RNA-seq library was prepared using KAPA RNA Hyper Prep kit (KAPA Biosystems), following the instruction (24,25). Resulting libraries were analyzed on an Agilent Bioanalyzer DNA 1000 chip (Agilent). Sequencing was performed on a Hiseq 2500 (Illumina) following the manufacturer's instructions.

### Calculation of differentially expressed gene

The raw sequence reads of the RNA-seq results were mapped onto each reference genome (Dataset 1) using Bowtie with the maximum insert size of 1000 bp, and two maximum mismatches after trimming 3 bp at the 3' ends (19). SAM output files generated by Bowtie were changed to BAM format using SAMtools. We analyzed the differential gene expression using the DESeq package (26). After the mapping, we count the number of reads that overlap each gene in the gff file, which contains the annotation information of each gene. Transcripts per million (TPM) value for each gene was calculated. By comparing the wild type and Δ*fur* strain, the degree of expression change level was calculated. The differentially expressed genes were defined as genes with expression value with log_2_ (fold change) ≥ 1.0 and *p*-value ≤0.05 or log_2_(fold change) ≤–1.0 and *P*-value ≤0.05. The gene expression profiling data were listed in Dataset 3.

### Pan-regulon analysis and functional characterization

Using the Metascope program, each peak and annotation information were visualized at the genome. For each peak, the nearest target genes or operons were identified as strain-specific regulon, based on the presence of differential expression, distance from the peak, and the promoter location from EcoCyc, BioCyc, and DOOR database (27–29). The number of strain-specific pan-regulons is listed in Table 2. All regulon genes were categorized into core (if they were shared across all strains), accessory (if they were shared across a subset of strains) and unique Fur regulon (if they are only in one strain). The comparisons of pan-genome, pan-binding, and pan-regulon are listed in Supplementary Tables S3–S6.

### Virulence factor (VF) analysis

Virulence factors were annotated by searching for amino acid sequence similarity against the curated set of genes extracted from the virulence factor database (VFDB) (30). Virulence factors were categorized based on the description of each curated gene into heme uptake, siderophore, transport, fimbriae, AAI/SCI-II and others. By performing one-tailed Fisher's exact test (Hypergeometric test) for virulence factor in Core, Accessory, and Unique category, the degree of enrichment was compared, and *P*-value ≤0.05 was considered significant.

### Clusters of orthologous groups (COGs) enrichment

Fur regulons were categorized according to their annotated COG database (31). Functional groups in core, accessory, and unique Fur-regulated genes were determined by COG categories.

## RESULTS

### Selection of strains from diverse phylogroups

We selected eight *E. coli* strains and one previously characterized reference strain for Fur regulon identification: K-12 MG1655 (the reference strain), W3110, Crooks, BL21(DE3), W, KO11FL, CFT073, 042, and Sakai, belonging to five phylogenetic groups (A, B1, B2, D and E). These eight strains were selected to represent the major phylogroups of *E. coli* as well as both laboratory strains and clinical isolates. W3110, Crooks, BL21(DE3), W and KO11FL are industrially relevant strains. CFT073, 042 and Sakai belong to enteroaggregative *E. coli* (EAEC), uropathogenic *E. coli* (UPEC), and enterohemorrhagic *E. coli* (EHEC), respectively (32,33). The general genomic features of those strains are in comparison with the reference strains (Supplementary Table S1). We found that the pathogenic strains (CFT073, 042, Sakai) have larger genomes compared to the non-pathogenic strains (W3110, Crooks, BL21(DE3)). This suggests that pathogenic strains have higher numbers of genes and operons as well as dynamic regulation.

### Characteristics of the fur regulon in the reference strain

**Figure 1.**
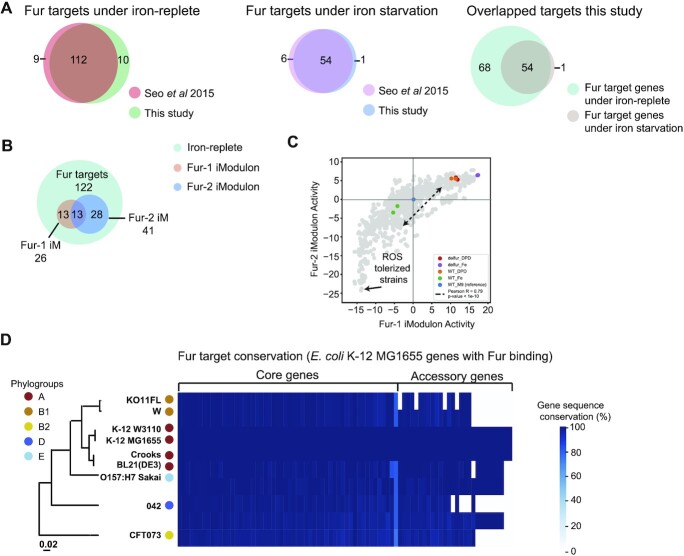
The characteristics of the Fur regulon in the MG1655 reference strain. (**A**) Comparison of previously reported target genes in MG1655. The ChIP-exo results from this study are very similar to what has been reported in the literature. (**B**) Overlaps between Fur targets under iron-replete, Fur-1 and Fur-2 iModulons. Genes reported to be in the two Fur iModulons (34) are all amongst the target genes found under iron replete conditions. Tables 3 and 4 have the COG categorization of the genes in these two iModulons. (**C**) The activity level (weighted sum of the expression level of the genes in an iModulon (iModulonDB.org)) of the Fur iModulons are correlated. Several conditions are highlighted in the figure including the reference conditions found in PRECISE 1K (36) (Blue), the wild type under iron starvation conditions (Orange), the wild type under iron replete conditions (Green), the *fur* deletion strain under iron-replete conditions (Purple), and under the iron starvation conditions (Red), and a few additional conditions of interest are highlighted (ROS tolerance strains with arrows) in the figure. Other datasets from PRECISE 1K (36) are shown in light gray. (**D**) The nucleotide sequence similarity between the Fur target genes in MG1655 and those found in the eight additional strains in this study. The conserved target genes in all nine strains are highly homologous. Not all of the eight additional strains have all the target genes found in MG1655. Even the non-conserved MG1655 target genes are highly homologous with the genes in the strains where they are also found.

We began our analysis by establishing the characteristics of the known Fur regulon in the MG1655 reference strain. We used our experimental protocols to determine the Fur regulon in MG1655 and then compared these results with the Fur regulon described in a previous study (17). As in the previous study, data was collected under iron replete and iron starvation conditions. The Fur binding sites determined in this study and the previous study showed a high degree of consistency (Figure 1A).

Fur is activated by binding to Fe^2+^ and thus the genome-wide binding site map differs between the iron replete and starvation growth conditions. Thus, we further compared the binding sites identified between the two conditions (Figure 1A). All but one of 55 Fur binding sites found under iron starvation conditions were also found under iron replete conditions. An additional 68 binding sites were found only under iron replete conditions. Thus, as expected, Fur actively regulates more genes in the presence of abundant iron (17).

New data analytic methods have been developed to analyze a large number of transcriptomes generated for a bacterial strain (34). This approach finds co-regulated sets of genes by using hundreds of expression profiles. Two sets of such coregulated genes are associated with Fur. These independently modulated sets of genes (iModulons) associated with Fur have genes found within the target genes that we identified in this study (Figure 1B). iModulons are a big data analog of regulons, computed in a top-down manner from the transcriptome composition under a variety of conditions (35).

iModulons consist of two things: their gene members and activity levels. The activity level indicates how active that set of genes is under different conditions (34). There are two iModoulons associated with Fur, called Fur-1/Fur-2 iModulons. Fur-1 mostly contains genes for siderophore synthesis and transport (Table 3). And the Fur-2 iModulon contains ferrous iron transport genes, as well as siderophore transport and hydrolysis systems (Table 4). We highlight the two iModulons activity levels over 1000 RNA-seq samples (36) from MG1655 (Figure 1C), and found these two iModulons show coordinated activity levels across over 1000 RNA-seq samples (36) from MG1655 (Figure 1C). It seems that the activities of these two iModulons trace out a trajectory, which captures the nonlinear effect of Fur on the composition of the transcriptome. Growing wild type strains under iron-rich conditions (green dots) results in lower Fur-2 iModulon activities, indicating lower ferrous iron transport activities in response to excess free iron availability.

Using this compendium of transcriptomes, we can locate the activity levels of the Fur iModulons under the iron replete and iron starvation conditions. The iron starvation condition is located close to the Fur deletion strain in the upper right corner in Figure 1C. The iron replete condition is located towards the ROS tolerized strains (via laboratory evolution) in the lower left corner of Figure 1C. The data set is normalized relative to the standard M9 condition located at the origin (0,0) of the plot. The expression profiles that we obtained under the iron-replete and iron-starvation conditions are thus well contextualized against this legacy compendium of MG1655 transcriptomes. The data obtained in this study on the reference strain is therefore highly consistent with various data in the literature (17).

We compared the genes in the Fur regulon in the MG1655 strain to the corresponding genes in the eight additional strains. In order to make functional assignments to genes, pangenome analysis requires a comparison of the sequences of corresponding open reading frames in the nine strains. We chose the cut-off sequence similarity 80% for the detailed analysis using BPGA (10) to cluster the protein sequences into core, accessory, and unique genomes (Table 1). The sequence similarity between the target genes of Fur in the MG1655 strain and those in the eight strains chosen is shown in Figure 1D.

**Table 1.** The pan-genome analysis generated by BPGA using 80% sequence identity

Phylogenetic group	Strains	Core gene	Accessory gene	Unique gene
A	K-12 MG1655	3058	982	18
A	W3110	3058	1297	36
A	BL21(DE3)	3058	1142	86
A	Crooks	3058	1240	140
B1	W	3058	1618	144
B1	KO11FL	3058	1515	8
B2	CFT073	3058	1117	612
D	042	3058	1412	588
E	Sakai	3058	1081	748

### Characterization of fur binding sites across different strains

With a well-characterized reference strain, we proceeded to identify the Fur binding sites in the eight additional *E. coli* strains. Using ChIP-exo, we enumerated the Fur target genes in each strain. We then compared the nucleotide sequences of the Fur target genes found in the eight strains to the target genes in *E. coli* K-12 MG1655 (Figure 1B). As observed, the target genes found in the eight strains were highly homologous to the target genes found in MG1655. We also compared the amino acid sequences of Fur in all nine strains, and found them to be identical (Supplementary Figure S1). Thus Fur and its target genes in the MG1655 Fur regulon are found to be highly conserved across all strains used in this study.

**Figure 2.**
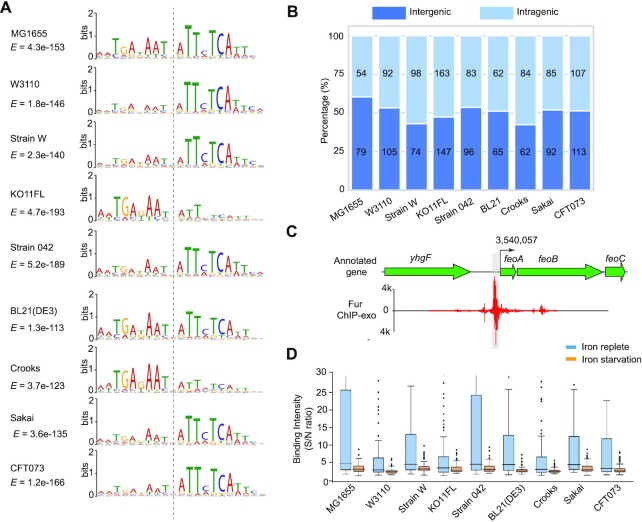
Characterization of Fur binding peaks. (**A**) A summary of the binding motifs in the nine strains under iron-replete conditions. The overall motif is highly conserved in the nine strains. E-value indicates the statistical significance of a motif discovered by MEME. (**B**) A summary of the genomic location of the binding peaks showing the number of peaks in the intragenic and intergenic regions. (**C**) Zoom-in binding site of Fur upstream of operon *feoABC* in *E. coli* K-12 MG1655. D) The average binding intensity of the Fur binding peaks under iron-replete and iron-starvation conditions. Fur binding is much more intense under iron-replete conditions, compared with iron-starvation conditions.

The ChIP-exo data identified 1322 binding sites across all nine strains. The strain-specific target genes are described in Dataset 2. We then determined the consensus DNA binding motif in all nine strains. Since the iron-replete and iron-starvation conditions differ in terms of the number of Fur binding peaks identified, we sought to determine if there are binding motif differences between iron-replete and iron-restricted conditions. First, under the iron-replete conditions, these motifs are highly conserved across the strains (Figure 2A). The consensus sequence in the Fur box contains TGAnAATnATTcTCA, which is similar to the consensus sequence previously described for *E. coli* ‘Fur box’ (37). Under the iron starvation conditions, we found the conserved sequence TTcTCA that corresponds to the second half of the consensus motif identified under iron-replete conditions (Supplementary Figure S2). It suggests that Fur has strong binding intensity at high Fe^2+^ levels. In contrast, Fur binds to weaker sites at low Fe^2+^ levels.

We found Fur binding peaks both in inter- and intra-genic regions (Supplementary Figure S3). There is a similar number of peaks found in both categories among all nine strains (Figure 2B). One expects to find binding peaks in the intergenic regions, especially in the promoter regions close to the -10/-35 boxes where the RNA polymerase binds. For example, Fur directly binds to the upstream of operon *feoABC* (Figure 2C) (38). The high number of binding peaks in coding regions can be examined in detail in the MG1655 strain, as extensive genome annotation data for this strain is available (39).

We searched for cases where the promoter of a gene overlaps with the coding region of the upstream gene in the reference strain. To investigate the promoter region, we further studied the RNA polymerase binding sites (RpoB) (17), and found that 43 binding sites may be located in the promoter region of the associated gene among the 57 peaks in a coding region in the MG1655 strain.

Finally, since Fur is activated by iron binding, we examined if the binding peaks were more intense for the iron-replete conditions. We found this to be the case for all nine strains (Figure 2D), showing stronger regulatory activity of Fur under iron replete conditions. The average intensity (S/N) under iron replete conditions and iron starvation conditions for nine strains are 28.02 and 3.27, respectively.

### The fur pan-regulon and its division into core, accessory, and unique regulons

**Figure 3.**
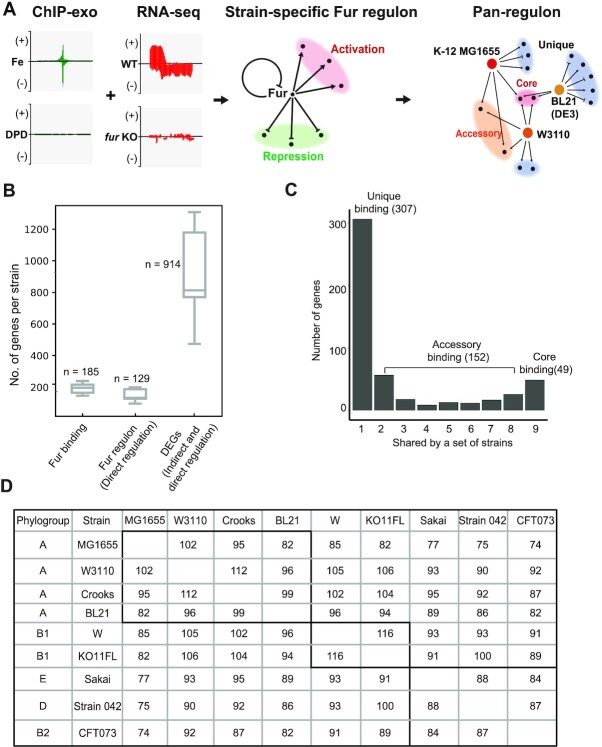
The workflow used to elucidate the Fur pan-regulon. (**A**) Schematic that shows the integration of ChIP-exo data and differential gene expression data (DEGs). If a target gene identified by a Fur binding peak shows differential expression between the wild type strain and the corresponding Fur deletion strain, that gene is assigned to the Fur regulon of that strain. Then the strain specific regulons are combined to generate a pan-regulon. (**B**) The number of Fur binding sites in the nine strains (average 185) and number of differentially expressed genes (average 129) directly regulated by Fur is shown. The average number of all differentially regulated genes is 914, including the 129 directly regulated genes by Fur. The full list of differentially expressed genes are found in Dataset 3. (**C**) Histogram that shows the number of strains with binding peaks in a promoter. We define the core binding to be the set of promoters where all nine strains have a binding peak detected. We define the unique binding to be the set of promoters where a binding peak is detected in only one strain. (**D**) Bi-directional Best Hit (BBH) homology relationships between the nine *E. coli* strains. Based on the phylogenetic tree, the BBH homology relationships showed that the same phylogroup *E. coli* strain shares similar binding sites compared to the strains from different phylogenetic groups.

Regulons are generally defined based on two properties. First, the identification of a binding peak of a transcription factor upstream from a gene (here via ChIP-exo), and second, determining expression differences of the target genes (here via RNA-seq) between the wild type strains and a strain where the transcription factor gene has been deleted. In most cases, the differential expression profiling is determined under a single growth condition. The integration of these two data types leads to a workflow shown in Figure 3A. The identification of the pan-regulon is then determined by comparing the regulons identified in each strain.

In the execution of this workflow, we need to tabulate the binding peaks, their corresponding target genes, and their gene expression (Figure 3B). If the expression of a target gene changes with the deletion of Fur, it means we have detected the direct regulation of Fur. The number of Fur binding sites found in the strains averages 185. Of those 185 binding sites, the number of differentially expressed genes averages 129, representing directly regulated target genes (Figure 3B). The directly regulated genes can, in turn, affect the expression of other genes. This is known as indirect regulation. We find that the average number of all differentially regulated genes is 914 across the nine strains, including the 129 genes directly regulated by Fur (Figure 3B).

The pan-regulon is defined based on the identification of the Fur binding sites across different strains. We used a publicly available method for binding peak identification (17), Once the peaks were identified in all the strains, we could compare the occurrence of binding sites across the nine strains (Figure 3C). Fur target genes are conceptually defined as the gene directly bound by Fur. The pan-binding is divided into core, accessory, and unique binding (Figure 3C). We found that the most common target genes (defined as core-binding genes) are involved in iron homeostasis, including inorganic ion transportation, and that accessory and unique regulon genes play diverse physiological roles, including macro-molecular (lipids, nucleotides, amino acids) transport, and metabolism. Meanwhile, we found there were still large amounts of unknown genes in the accessory/unique bindings, which implied the novel regulation of Fur.

To explore Fur binding across different phylogroups, we further compared the Fur binding sites between each strain, and we observed that highly related strains showed a higher degree of Fur site similarity (Figure 3D and Supplementary Figure S4). For example, in phylogroup A (K-12 MG1655, W3110, Crooks, BL21), MG1655 and W3110 shared 102 Fur binding sites and Crooks and BL21 shared 99. However, MG1655 only shared 74 binding sites with CFT073, a pathogenic strain that is phylogenetically distant from the reference strain. Overall, these results suggest that Fur may play similar regulatory roles in the closely related strains.

### Detailing the genes found in the core, accessory and unique fur regulons

**Figure 4.**
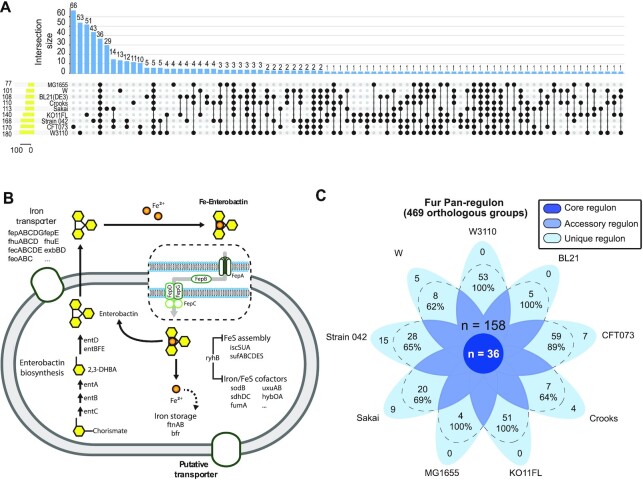
The division of the Fur pan-regulon into core, accessory, and unique regulons. (**A**) An UpSet plot showing shared and unique regulon of nine *E. coli* strains. Connected dots represent the intersections of overlapping orthologs with the vertical black bars above showing the number of orthogroups in each intersection. The value above the blue chart (top panel) shows the number of shared or unique regulon corresponding to the connected dots below. The value nearby the yellow chart (left panel) shows the total number of Fur regulon in *E. coli* strains. (**B**) The function of differentially expressed Fur target genes found in all nine strains (*n* = 36, full list of gene identities and their expression are shown in Supplementary Table S7). This is the core Fur regulon representing the common target between all the strains, with functions in inorganic transport and metabolism. (**C**) A Venn diagram of the genes in the Fur regulons in nine strains schematically showing the core, the accessory, and the unique regulons. The unique regulon is further divided with a dashed line showing: (i) genes unique to a strain that are regulated by Fur, and (ii) genes that are not unique to a strain that are regulated by Fur in that strain and not any of the other nine strains.

To study the strain-specific Fur regulons, we use an UpSet plot showing differentially expressed Fur target genes specific to a single strain (first nine bars in Figure 4A). These genes constitute Fur regulatory targets only found in one strain - the unique regulon.

The remaining bars in the UpSet plot show differentially expressed Fur target genes found in two or more of the strains, but not in all nines (Figure 4A). We call this the accessory Fur regulon, analogous to the definition used in pangenomics of an accessory genome. This set of target genes is the core Fur regulon, representing the common target between all the strains (Figure 4B).

Furthermore, we depicted the absence-presence-variation of the Fur pan-regulon in *E. coli* (Dataset 4). Absence-presence-variation (APV) of regulon, refers to the absence, presence, or variation of target genes at the transcriptional level. It is an important genetic feature to discover differential regulation across diverse strains. For the core regulon, all of the target genes are present across the strains (Supplementary Figure S5, Supplementary Table S2). Our results showed that the majority of core regulon have similar transcriptional regulation patterns with the exception of for *ydiE* (black arrowhead in Supplementary Figure S5). Specifically, core regulons were up-regulated upon the deletion of gene *fur*, but only core regulon gene *ydiE* with the unknown function, was down-regulated in *E. coli* BL21.

Finally, we can graphically summarize the segregation of the strain-specific Fur regulons. A Venn diagram of the genes in the Fur regulons in the nine strains schematically showing the core, accessory, and unique regulons is shown in Figure 4C. The number of genes in each category is shown. The full list of genes in each category is summarized in Dataset 4.

The unique regulon can be further divided into two categories of target genes (dashed line, Figure 4C): (i) genes unique to a strain that are regulated by Fur, and (ii) genes that are not unique to a strain that are regulated by Fur in that strain and not any of the other nine strains. Here. we show that these unique genes are under Fur regulation. Upon deletion of Fur, these genes then exhibit changes in expression. These results thus show that strains can harbor unique genes that are regulated by Fur, though additional regulation of these genes by other TFs is possible.

### Functional attributes of the pan-fur regulon

After revealing the Fur regulons in nine strains, and comparing their molecular, genetic and regulatory properties, we examined the physiological and functional characteristics that can be attributed to differences in strain specific regulons.

**Figure 5.**
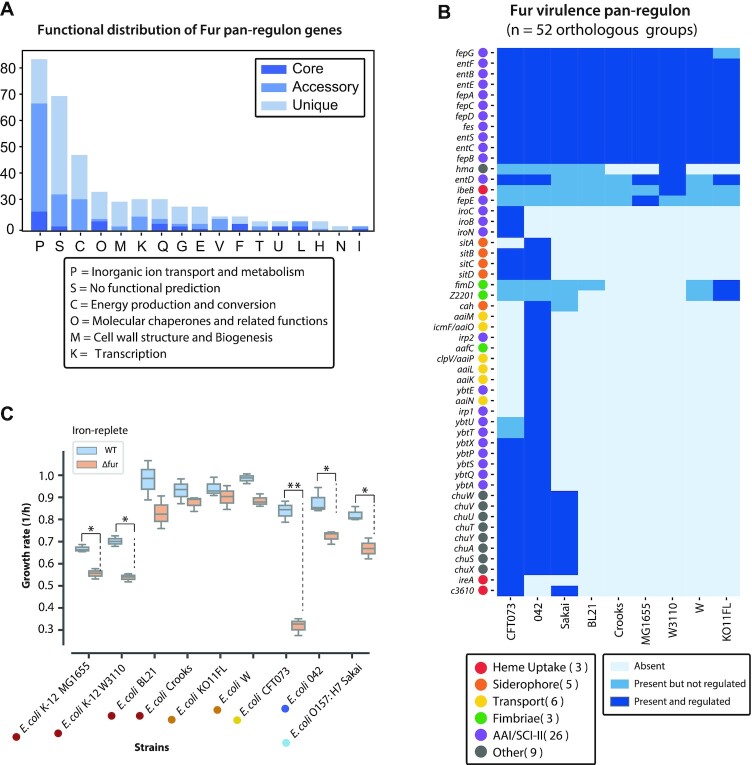
Functional attributes of the pan-Fur regulon. (**A**) COG functional characterization of the genes in the core, accessory, and unique Fur regulons. The most common gene function in the core is inorganic transport and metabolism. Other gene functions are distributed throughout the COG categories. Only their class abbreviations are used here, their corresponding class descriptions are listed: **P**, Inorganic ion transport and metabolism; **S**, Function unknown; **O**, Post-translational modification, protein turnover, chaperones; **M**, Cell wall/membrane/envelope biogenesis; **K**, Transcription; **Q**, Secondary metabolite biosynthesis, transport and catabolism; **G**, Carbohydrate transport and metabolism; **E**, Amino acid transport and metabolism; **V**, Defense mechanisms; **F**, Nucleotide transport and metabolism; **T**, Signal transduction mechanisms; **U**, Intracellular trafficking, secretion, and vesicular transport; **L**, Replication, recombination and repair; **H**, Coenzyme transport and metabolism; **N**, Cell motility; **I**, Lipid transport and metabolism; (**B**) The distribution of virulence factors in the Fur pan-regulon. The pathogenic strains (clinical isolates) contain many more virulence factors than the commonly used laboratory strains. (**C**) Physiological consequences of Fur deletion in the nine strains. For four strains (MG1655, W3110, CFT073, 042), there is a statistically significant growth rate reduction resulting from *fur* gene deletion.

First, we carried out Clusters of Orthologous Groups (COG) functional characterization of the genes in the core, accessory, and unique Fur regulons of the strains (Figure 5A). Several observations can be made. The most common gene functions found in the core regulon are related to inorganic transport and metabolism. This is consistent with the basic regulatory functions attributed to Fur. Other functional gene attributes seem to be distributed throughout the COG categories.

Second, since we have pathogenic and laboratory strains amongst the nine strains, we examined the distribution of virulence factors in the Fur pan-regulon (Figure 5B). The pathogenic strains (clinical isolates) contain many more virulence factors than the commonly used laboratory strains. We utilized the pan-regulon to explore the effect of Fur on the regulation of virulence. In total, virulence factors constituted 11% (52 out of 469) of the pan-regulon, of which only nine were commonly regulated by Fur across all strains, including operon *fepABCD* (encoding protein that binds and transports ferric enterobactin (ferric enterochelin (27)), and operon *entBCEFS* involved in the enterobactin biosynthesis pathway (40) (Figure 5B). Incidentally, the three pathogenic strains, (strains 042 (*n* = 40), CFT073 (*n* = 31), and Sakai (*n* = 19)), had the largest numbers of Fur regulated virulence factors among the nine strains. Specifically, eight heme uptake and utilization genes (*chuASTUVWXY*) were uniquely shared and regulated by Fur in the pathogenic strains (41), while *sitABCD* (mediating metal ion transport) and *ybtAEPQSTUX* (mediating siderophore biosynthesis) were regulated in two of the three pathogenic strains (42,43). Strain 042 (an EAEC strain) stood out as being the only strain having *aaiKLMNOP* (a putative type IV secretion system) in its Fur regulon (30), while strain CFT073 (a UPEC strain) was the only strain to include *iroBCN* (mediating salmochelin synthesis) (44). Importantly, when strains were clustered according to their Fur regulon, the three pathogenic strains clustered together while the nonpathogenic strains clustered according to their phylogenetic background. This difference, and the active regulation of many of these target genes, suggest that Fur plays an important role in virulence.

Third, we examined the growth properties of the nine wild type strains and their corresponding *fur* deletion strains (Figure 5C). For four of the nine strains (MG1655, W3110, CFT073, 042), there is a statistically significant growth rate reduction resulting from *fur* gene deletion. For the remaining five strains, no significant changes were observed as a result of deleting *fur* gene.

Fourth, we investigated the production of siderophore and found that *fur* deletion strains generated a high level of siderophore compared to the wide type, when cells grew to the stationary phase (Supplementary Figure S6), which were similar to the phenotype from *fur* deletion mutants in other bacteria (45). Our results suggested that most of the target genes relevant to siderophore synthesis belong to the core regulon and were highly upregulated upon the deletion of Fur across the strains (Supplementary Table S8).

Finally, we studied the influence of Fur on antibiotic resistance, and found that *fur* deletion pathogenic strains showed antibiotic sensitivity (Supplementary Figure S7). Previous studies showed that loss of Fur facilitated the evolution of ciprofloxacin resistance in *Escherichia coli* BW25113, and found expression change of a set of genes (*tar, fliA, fiu, mntH, amtB, citC, entC, entE, wzc, yfiL, yjjZ*) associated with adaptive resistance (46), but there was no evidence to show the regulation of Fur on those target genes in the strain. Here, we mapped related regulatory networks to pan-regulons and found *fiu*, *mntH*, *entC*, *entE* and *yjjZ* genes belong to the core regulon (Supplementary Figure S8). Further, the expression of those genes were upregulated upon the deletion of Fur (Supplementary Table S9). These results demonstrated that Fur and its core regulon are directly involved in control of antibiotic resistance in the *E. coli* strains.

## DISCUSSION

The function of many global transcription factors have been elucidated using laboratory model strains. This information is summarized for the MG1655 reference *E. coli* strain in the widely used RegulonDB database (47). We, and others, have diligently been elucidating individual regulons for a growing list of transcription factors (17,35,48–60). Thus, comprehensive information is available about the transcriptional regulatory network in the MG1655 strain. A fundamental question that remains is how generalizable are results from a model strain to other strains of the *E. coli* species? We thus selected eight *E. coli* strains, laboratory and clinical, and elucidated their regulons using established genome-wide profiling methods. As a result, we were able to compare strain-specific regulons (Table 2). A comparison of these strain-specific regulons resulted in the elucidation of the first pan-regulon for a bacterial transcription factor.

**Table 2.** The number of Fur strain-specific regulon across the *E. coli* strains

Strains	Core regulon	Accessory regulon	Unique regulon	Total Fur regulon
K-12 MG1655	36	37	4	77
W3110	36	91	53	180
BL21(DE3)	36	67	5	108
Crooks	36	63	11	110
W	36	52	13	101
KO11FL	36	53	51	140
CFT073	36	68	66	170
042	36	89	43	168
Sakai	36	48	29	113

Note: cut-off value (log_2_ fold change ≥1.0, or ≤–1.0 and *P*-value < 0.05) for the differentially expressed genes

The criteria for defining strain-specific regulons and pan-regulons are under debate, and the discussion is only intensifying as more genome-wide datasets are becoming available. In this study we identify core regulons that are common to any group of strains in our study, to determine whether genes specific to a particular taxon exist and to investigate their potential role in adaptation of bacteria to their specific niche.

Based on the data set generated, we define a pan-regulon consisting of 469 target genes that includes a union of all target genes of Fur in all nine *E. coli* strains studied. In analogy to pangenomic studies (61,62), we then divided the pan-regulon into the core regulon (the Fur regulated genes found in all the strains, *n* = 36), the accessory regulon (for regulated genes found in two to eight strains, *n* = 158), and the unique regulon (Fur regulated genes found in one strain, *n* = 275).

We found a relatively small set of Fur regulated genes common to all nine strains, but a large number of regulatory targets unique to a particular strain. The set of common regulatory target genes is consistent with what is known about the basic functions of Fur in regulation of iron metabolism. The core regulon consists of iron uptake-related target genes, indicating the direct regulation of iron uptake is highly conserved across all nine strains. The set of core regulon genes belong to two Fur iModulons whose activities have a non-linear relationship, previously described in detail (Figure 1C, Tables 3 and 4). The high number of unique regulatory targets of Fur shows a somewhat surprising and significant diversity in Fur regulation amongst the nine *E. coli* strains. This presumably reflects diverse niche specification and strain history. Interestingly, many of the genes that are uniquely regulated by Fur in a strain are also genes unique to that strain.

**Table 3.** The COG categorization of the genes in Fur-1 iModulons

Fur-1 iModulon COGs	No. of genes	Gene name
Inorganic ion transport and metabolism	11	*fepA, fiu, cirA, fes, fhuE, fepC, ydiE, fepG, fhuB, fhuD, fhuC*
Secondary metabolites biosynthesis, transport and catabolism	4	*entB, entE, entH, entF*
Coenzyme transport and metabolism	2	*entC, entD*
Nucleotide transport and metabolism	2	*nrdE, nrdF*
Amino acid transport and metabolism	1	*entS*
Lipid transport and metabolism	1	*entA*
Signal transduction mechanisms	1	*ybiI*
Post-translational modification, protein turnover, and chaperones	1	*sufD*
Function unknown	3	*ybdZ, ybiX, yncE*

**Table 4.** The COG categorization of the genes in Fur-2 iModulons

Fur-2 iModuon COGs	No. of henes	Gene name
Inorganic ion transport and metabolism	21	*bfd, fhuA, fepD, feoA, fecR, ydiE, fes, feoB, fepG, fepB, yojI, fepC, nfeF, fhuC, fhuD, yddA, sodA, efeO, yoeA, mntP, ftnA*
Intracellular trafficking, secretion, and vesicular transport	4	*exbB, exbD, rcnA, tonB*
Transcription	2	*fecI, feoC*
Secondary metabolites biosynthesis, transport and catabolism	2	*entB, entH*
Energy production and conversion	2	*ydhV, ydhY*
Amino acid transport and metabolism	1	*entS*
Lipid transport and metabolism	1	*entA*
Coenzyme transport and metabolism	1	*entC*
Nucleotide transport and metabolism	1	*nrdI*
Post-translational modification, Protein turnover, chaperons	1	*nrdH*
No COG annotation	1	*efeU*
Function unknown	4	*fhuF, yncE, ybaN, ybdZ*

Thus, there are genes that are members of both the unique genome and the unique Fur regulon found in a strain. This diversity likely results in physiological differences between strains that need further elucidation on a per strain basis. Such elucidation of strain specific properties will be especially important in pathogenic strains as these differences may affect their virulence and pathology, and thus lead to development of specialized treatment modalities for specific strains. Such developments would equate to personalized medicine in infectious disease, but personalized for the pathogen.

## DATA AVAILABILITY

The new dataset of ChIP-exo and RNA-seq (eight *E. coli* strains) generated in this study have been deposited to the Gene Expression Omnibus (GEO) with the accession number of GSE150240 and GSE150501, respectively. The new ChIP-exo data for the reference strain MG1655 is deposited to GSE223483. The RNA-seq data for the reference strain MG1655 is available from GSM54900 (GSM1326347, GSM1326348, GSM1326351 and GSM1326352).

## Supplementary Material

gkad253_Supplemental_Files
