# Supplementary material for: The Escherichia coli Fur pan-regulon has few conserved but many unique regulatory targets
Source: Nucleic Acids Res. 2023 Apr 7;51(8):3618–30. doi: 10.1093/nar/gkad253 (PMC10164565; doi:10.1093/nar/gkad253)
Supplement: gkad253_Supplemental_Files [file gkad253_supplemental_files.zip › SI materials for pan-Fur regulon.pdf]

The *Escherichia coli* Fur pan-regulon has few conserved but many unique regulatory targets

Dataset 1 The strains used in this study
Dataset 2 The genome-wide binding event of Fur under iron-replete and iron-restriction conditions
Dataset 3 The gene expression profiling of *fur* knockout strains across nine *E. coli* under iron-replete conditions
Dataset 4 The pan-regulon results across nine *E. coli* strains

Supplemental materials

E. coli Fur pan-regulon has few conserved but many unique regulatory targets

The pan regulon of Fur is composed of 469 genes across nine *E. coli* strains (Dataset 4), including 36 core regulon, 158 accessory regulon, and 275 unique regulon, which are listed in the sheet "Pan-regulon genes". The "locus_tag or genes" corresponding to "cluster_group" are listed in the sheet "Locus_tag & Gene_name".

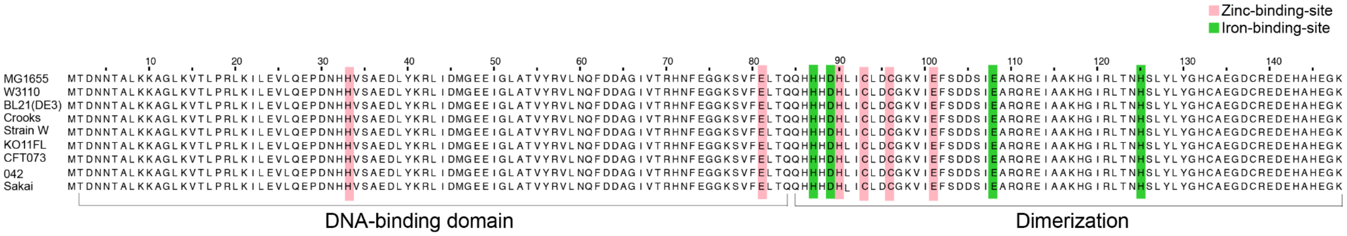

Supplementary figure 1 The amino acid sequence of Fur is highly conserved across nine *E. coli* strains.

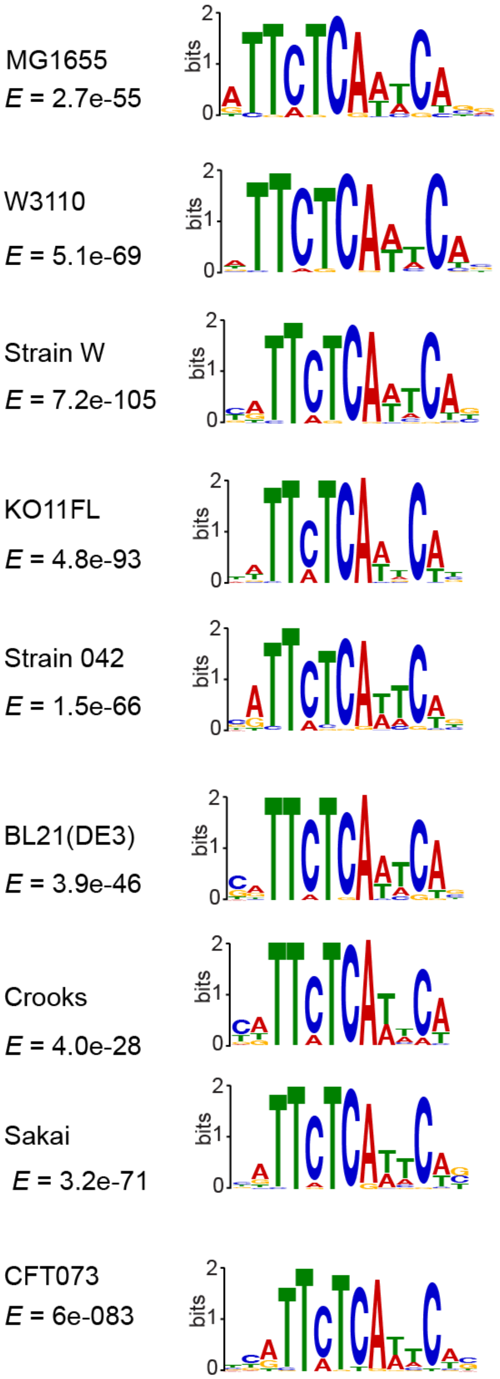

Supplementary figure 2 The consensus motif of Fur-box under iron-deplete conditions in all nine *E. coli* strains.

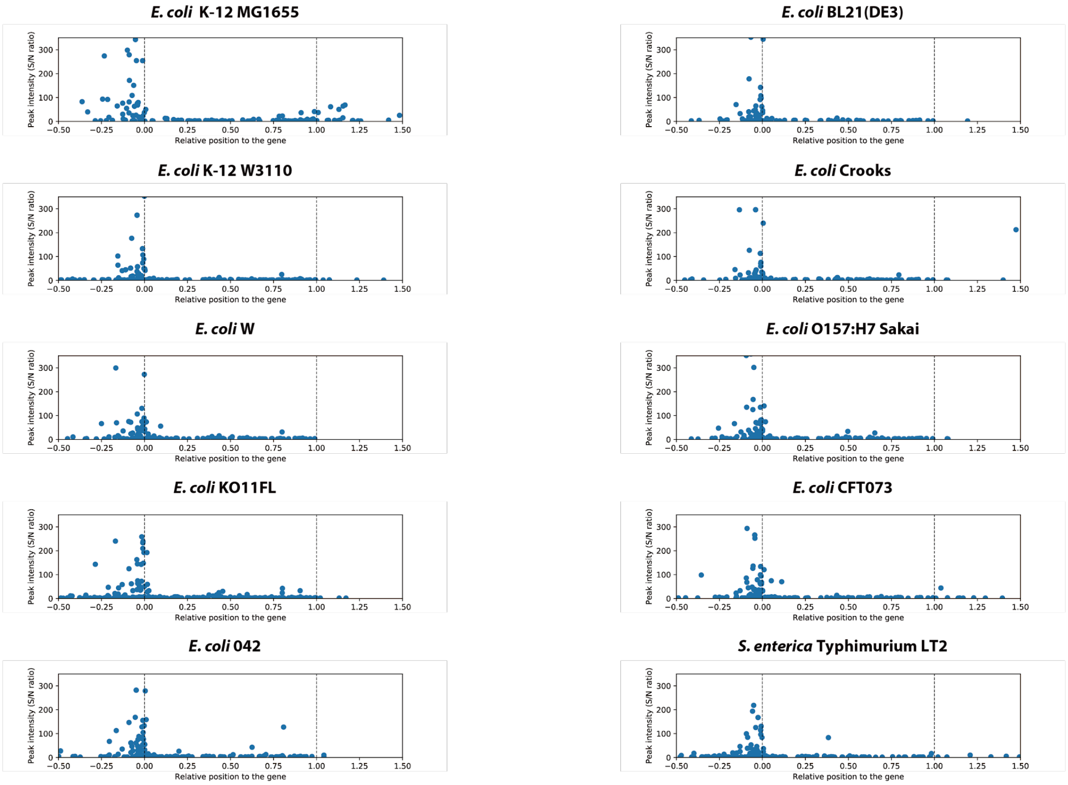

Supplementary figure 3 The results of the ChIP-exo analysis are expressed as scatter plots. This is the result of an iron-replete condition. The Y-axis is the value of the binding intensity of each peak. 0 on the X-axis represents the start site of the translation start site, and 1 represents the end site of the gene. The relative position of peaks relative to each gene was designated as x-value. Negative values indicate their upstream or promoter position.

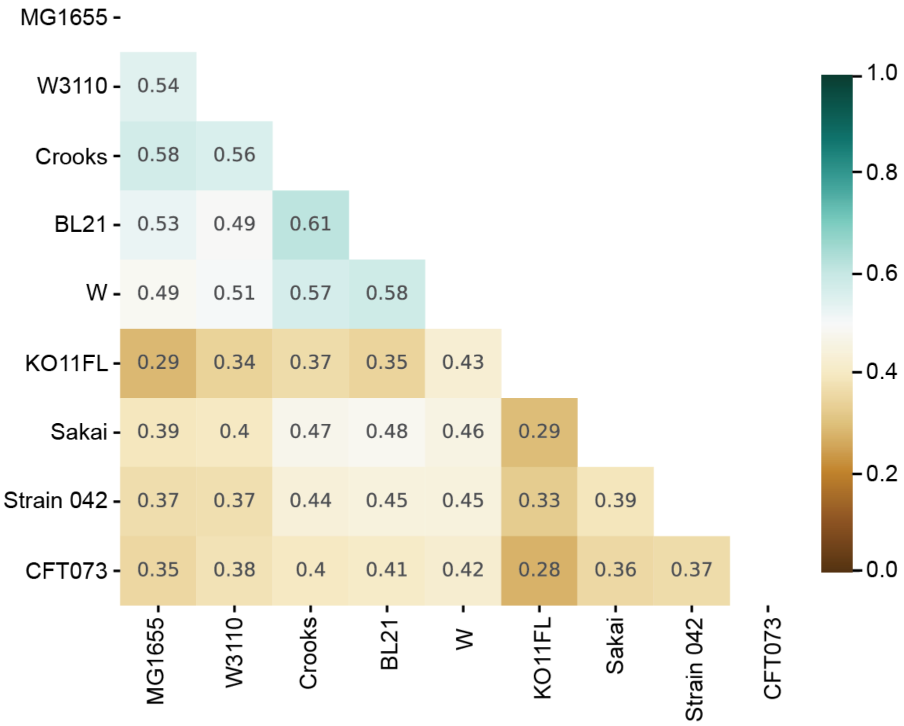

Supplementary figure 4 Bi-directional Best Hit (BBH) homology relationships between the nine *E. coli* strains. Heatmap showed the Jaccard similarity index of Fur binding sites between two closely related strains. The Jaccard similarity is calculated by (number of shared bindings) / (the number of bindings in either strains). If two strains share the exact same bindings, their Jaccard similarity index will be 1. Conversely, if they have no bindings in common then their similarity will be 0.

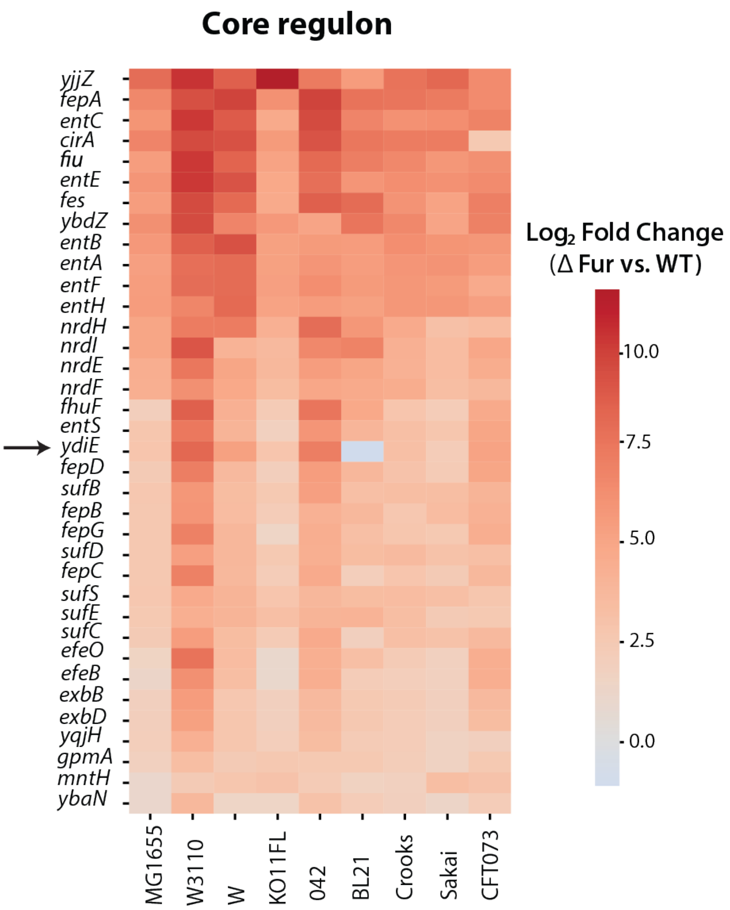

Supplementary figure 5 Heatmap showing expression of genes belonging to the core regulon. The arrowhead points out the gene *ydiE* in BL21. The color represents the $\log_2$ (fold change of *Δfur* vs. WT).

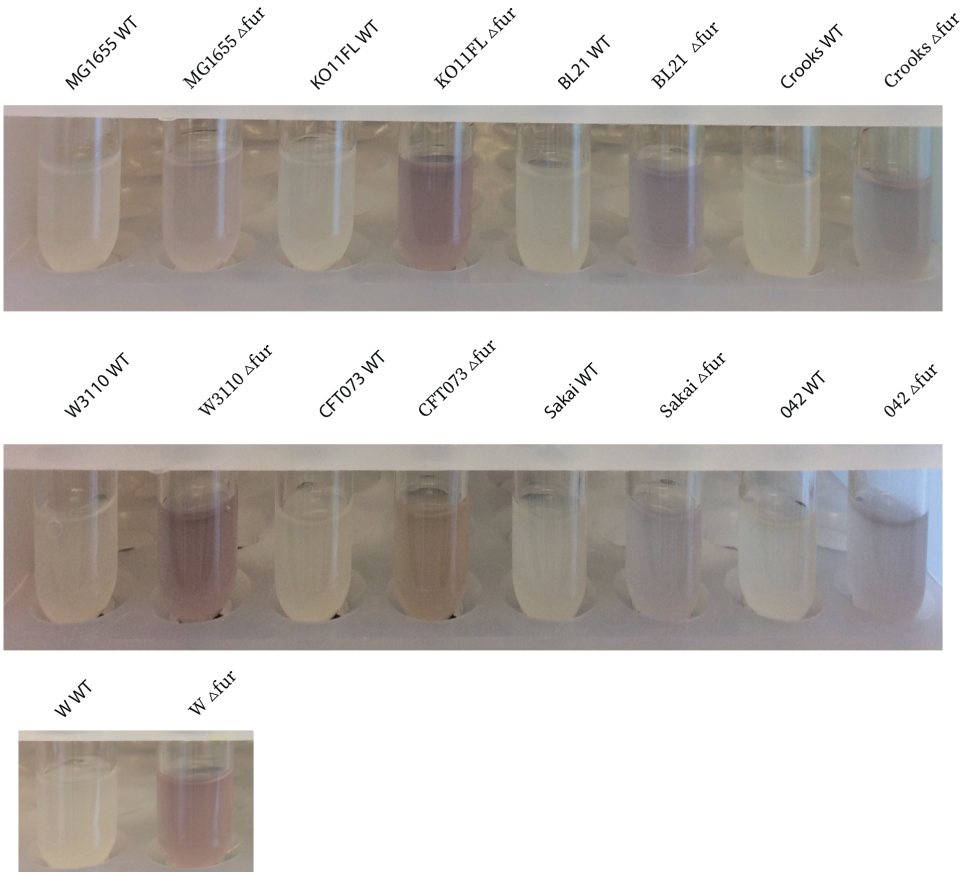

Supplementary figure 6 Detection of siderophore production for WT and fur knockout strains.

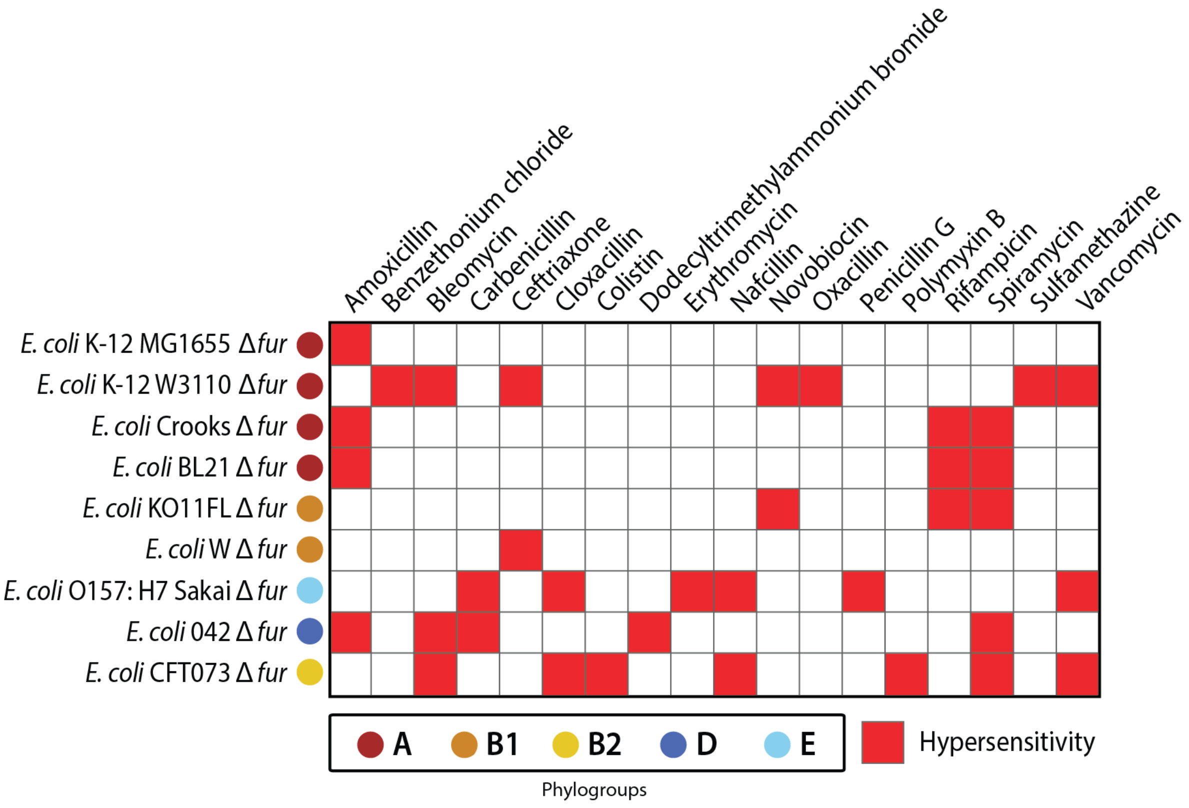

Supplementary figure 7 Antibiotic resistance profile according to phenotypic microarray (PM) plates.

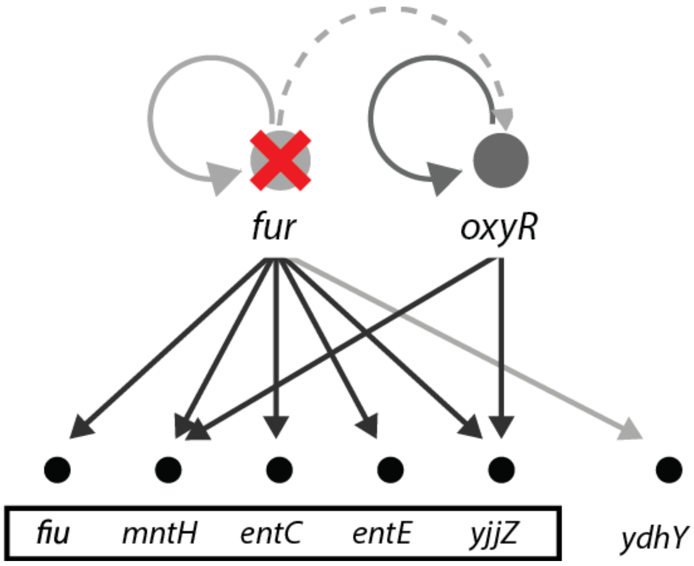

Supplementary figure 8 The regulatory network of Fur pan-mediated regulation to the antibiotic response. Arrows indicate the direction of the regulation (i.e. Fur directly regulates *fiu*).

Supplementary Table 1 General genomic features of 8 representative *E.coli* strains in comparison with K-12 MG1655

Strain	Phylogroup	Size (bp)	No. of genes	Gene density (genes/Kbp)	No. of contigs	Accession No.	Pathotype, serotype, other characteristics
K12 MG1655	A	4639675	4497	0.9692	1	NC_000913.2	Commensal, K12
W3110	A	4646332	4793	1.0316	1	NC_007779.1	Commensal, K12
Crooks (ATCC8739)	A	4746218	4827	1.0170	1	NC_010468.1	K12 derivative
BL21(DE3)	A	4558953	4700	1.0309	1	NC_012971.2	Commensal, strain
W	B1	5008864	5195	1.0372	3	NC_017635.1 /NC_017636.1 /NC_017637.1	
KO11FL	B1	5027172	5259	1.0461	2	NC_017660.1 /NC_017661.1	
CFT073	B2	5231428	5421	1.0362	1	NC_004431.1	UPEC, O6:K2:H1
042	D	5355323	5580	1.0420	2	NC_017626.1 /NC_017627.1	EAEC, O44: H18
Sakai	E	5594477	5446	0.9735	3	NC_002695.1 /NC_002127.1 /NC_002128.1	EHEC, O157: H7

Supplementary Table 2 The change of expression level for the core regulon in *E. coli* strains (the number of each element represents the $\log_2$ fold change of *Δfur* vs. WT for each gene, rounded to two decimal places)

	K-12 MG1655	W3110	W	KO11FL	042	BL21(DE 3)	Crooks	Sakai	CFT073
yjjZ	7.91	10.25	8.49	11.32	7.28	5.54	7.67	8.12	6.41
fepA	6.60	9.19	9.69	6.15	9.62	7.59	7.49	7.27	6.42
entC	5.98	9.99	8.70	4.73	9.39	6.77	6.08	6.32	6.89
cirA	6.73	9.40	9.17	5.61	9.01	7.46	7.18	7.19	2.55
fiu	5.47	10.03	8.35	5.19	8.04	7.09	6.53	5.83	6.20
entE	5.93	9.99	9.09	4.84	7.81	5.90	6.30	6.14	6.41
fes	5.42	9.39	8.07	4.71	8.50	7.95	6.06	5.18	7.02
ybdZ	6.19	9.40	6.73	5.70	5.07	7.58	6.61	5.09	6.95
entB	5.68	8.47	9.12	5.22	5.55	5.52	6.34	5.97	5.77
entA	5.35	7.79	7.94	5.21	5.86	5.42	5.84	6.05	5.43
entF	5.43	7.89	7.96	5.38	6.35	5.63	5.78	5.62	4.64
entH	5.53	6.66	8.07	5.19	5.54	5.24	5.78	5.75	5.39
nrdH	5.04	7.15	7.19	4.32	7.91	5.80	4.64	3.14	3.51
nrdI	4.97	8.94	4.11	3.66	6.61	6.84	4.27	3.53	4.94
nrdE	4.52	7.46	4.99	3.72	5.45	4.99	4.22	3.49	4.55
nrdF	4.43	6.25	4.82	3.41	4.91	4.70	4.53	3.31	3.78
fhuF	2.02	8.49	4.33	2.33	7.52	4.79	2.84	2.24	4.71
entS	2.75	7.42	4.07	1.83	5.86	3.99	3.18	2.78	5.02
ydiE	2.87	8.14	5.24	2.91	7.08	-1.10	3.20	2.23	5.16
fepD	2.47	7.13	3.67	2.05	5.45	3.83	2.98	2.36	4.99

sufB	2.72	5.79	3.46	2.50	5.36	3.24	3.33	3.35	4.06
fepB	2.62	5.86	3.51	2.23	4.19	3.77	2.69	3.54	4.19
fepG	2.61	6.91	3.82	1.45	4.45	3.21	2.82	2.53	4.48
sufD	2.68	5.39	3.87	2.54	4.38	3.42	3.57	3.11	3.23
fepC	2.67	6.94	3.78	2.25	4.65	2.06	2.88	2.47	3.74
sufS	2.73	4.63	4.01	2.92	3.88	3.44	3.51	3.20	2.81
sufE	2.58	4.37	4.00	3.22	4.06	4.10	3.33	2.46	2.52
sufC	2.46	5.42	3.44	2.35	4.66	1.86	3.25	3.05	3.66
efeO	1.55	7.67	3.51	1.06	4.37	3.21	2.35	1.82	4.52
efeB	1.30	6.25	3.32	1.02	4.50	2.27	2.12	1.78	4.49
exbB	1.87	5.53	2.71	1.95	3.66	2.55	2.32	1.86	3.61
exbD	1.97	5.27	2.81	1.89	3.59	2.68	2.22	1.91	3.44
yqjH	2.10	4.24	2.75	2.11	3.38	2.31	2.25	1.63	1.89
gpmA	1.77	3.33	2.45	2.64	2.52	2.57	2.20	1.65	2.59
mntH	1.16	2.49	2.79	2.99	2.33	1.72	1.85	3.30	2.93
ybaN	1.16	3.79	1.47	1.44	3.11	2.23	1.95	1.33	2.26

Supplementary Table 3 Comparison of Pan-genome and pan-binding

**Pan-genome**	**No. of genes**	**Pan-binding**	**No. of binding**
Core genome	3032	Core binding	49
Accessory genome	2289	Accessory binding	152
Unique genome	1791	Unique binding	307

Supplementary Table 4 Composition of pan-genome and pan-binding

**Pan-genome**	**No. of genes**	**Pan-binding**	**No. of binding**
Core genome	3032	Core binding	49
		Accessory binding	96
		Unique binding	15
		No binding	2872
Accessory genome	2289	Accessory binding	56
		Unique binding	166
		No binding	2067
Unique genome	1791	Unique binding	126
		No binding	1665

Supplementary Table 5 Comparison of Pan-genome and pan-regulon

Pan-genome	No. of genes	Pan-regulon	No. of regulon
Core genome	3032	Core regulon	36
Accessory genome	2289	Accessory regulon	158
Unique genome	1791	Unique regulon	275

Supplementary Table 6 Composition of pan-genome and pan-regulon

Pan-genome	No. of genes	Pan-regulon	No. of regulon
Core genome	3032	Core regulon	36
		Accessory regulon	88
		Unique regulon	25
		N/A	2883
Accessory genome	2289	Accessory regulon	70
		Unique regulon	110
		N/A	2109
Unique genome	1791	Unique regulon	140
		N/A	1651

Note, N/A denotes not available

Supplementary Table 7 The gene expression (values in Log_2FC) of Fur pan-regulon involved in iron metabolism, transport, and storage from *fur* knockout strains

**Category**	**Function**	**Representative gene**	***E. coli* K-12 MG1655**	***E. coli* K-12 W3110**	***E. coli* W**	***E. coli* KO11FL**	***E. coli* 042**	***E. coli* BL21(DE3)**	***E. coli* Crooks**	***E. coli* O157:H7 Sakai**	***E. coli* CFT073**
Core regulon	Fe transport	*cirA*	6.73	9.40	9.17	5.61	9.01	7.46	7.18	7.19	2.55
Core regulon		*efeO*	1.55	7.67	3.51	1.06	4.37	3.21	2.35	1.82	4.52
Core regulon		*efeB*	1.30	6.25	3.32	1.02	4.50	2.27	2.12	1.78	4.49
Accessory regulon		*feoA*	1.16	2.40	2.40	4.17	0.00	1.59	0.00	0.00	1.13
Accessory regulon		*feoB*	0.00	1.53	1.96	3.64	0.00	1.60	0.00	0.00	0.00
Core regulon		*fepD*	2.47	7.13	3.67	2.05	5.45	3.83	2.98	2.36	4.99
Core regulon		*fepG*	2.61	6.91	3.82	1.45	4.45	3.21	2.82	2.53	4.48
Core regulon		*fepC*	2.67	6.94	3.78	2.25	4.65	2.06	2.88	2.47	3.74
Accessory regulon		*fhuA*	1.09	5.00	2.18	0.00	3.10	1.97	1.07	1.40	3.93
Accessory regulon		*fhuC*	0.00	3.09	2.43	0.00	2.76	1.21	1.36	0.00	3.04
Accessory regulon		*fhuD*	1.07	3.14	2.51	0.00	3.03	2.11	1.35	1.21	2.44
Accessory regulon		*fhuB*	0.00	3.28	2.58	0.00	1.32	1.84	1.12	0.00	2.29

Accessory regulon		*fhuE*	5.02	6.15	6.14	3.89	N/A	5.61	5.29	8.38	3.02
Core regulon		*fhuF*	2.02	8.49	4.33	2.33	7.52	4.79	2.84	2.24	4.71
Core regulon		*fiu*	5.47	10.03	8.35	5.19	8.04	7.09	6.53	5.83	6.20
Accessory regulon		*ybiX*	5.21	7.12	5.71	4.47	6.79	5.94	5.83	4.73	N/A
Core regulon		*exbB*	1.87	5.53	2.71	1.95	3.66	2.55	2.32	1.86	3.61
Core regulon		*exbD*	1.97	5.27	2.81	1.89	3.59	2.68	2.22	1.91	3.44
Accessory regulon		*tonB*	1.62	4.81	2.25	1.58	3.64	2.98	2.15	N/A	3.16
Core regulon	Metabolism	*gpmA*	1.77	3.33	2.45	2.64	2.52	2.57	2.20	1.65	2.59
Accessory regulon		*adhP*	0.00	1.27	1.18	N/A	0.00	0.00	1.04	2.20	0.00
Accessory regulon		*yddA*	3.06	6.93	3.96	N/A	4.81	3.95	2.09	1.87	3.44
Accessory regulon		*yddB*	2.95	3.99	3.80	N/A	3.87	2.72	2.30	2.01	2.90
Core regulon		*nrdH*	5.04	7.15	7.19	4.32	7.91	5.80	4.64	3.14	3.51
Core regulon		*nrdI*	4.97	8.94	4.11	3.66	6.61	6.84	4.27	3.53	4.94
Core regulon		*nrdE*	4.52	7.46	4.99	3.72	5.45	4.99	4.22	3.49	4.55

Core regulon		*nrdF*	4.43	6.25	4.82	3.41	4.91	4.70	4.53	3.31	3.78
Accessory regulon	Fe-Stores	*bfd*	N/A	7.30	3.58	3.08	5.52	3.11	2.42	2.27	5.23

Note, N/A denotes not available

Supplementary Table 8 The gene expression (values in Log_2FC) of part of Fur pan-regulon involved in siderophore synthesis and export from *fur* knockout strains

**Category**	**Representative gene**	***E. coli* K-12 MG1655**	***E. coli* K-12 W3110**	***E. coli* W**	***E. coli* KO11FL**	***E. coli* 042**	***E. coli* BL21(DE3)**	***E. coli* Crooks**	***E. coli* O157:H7 Sakai**	***E. coli* CFT073**
Core regulon	*entC*	5.98	9.99	8.70	4.73	9.39	6.77	6.08	6.32	6.89
Core regulon	*entE*	5.93	9.99	9.09	4.84	7.81	5.90	6.30	6.14	6.41
Core regulon	*entB*	5.68	8.47	9.12	5.22	5.55	5.52	6.34	5.97	5.77
Core regulon	*entA*	5.35	7.79	7.94	5.21	5.86	5.42	5.84	6.05	5.43
Core regulon	*entH*	5.53	6.66	8.07	5.19	5.54	5.24	5.78	5.75	5.39
Core regulon	*fes*	5.42	9.39	8.07	4.71	8.50	7.95	6.06	5.18	7.02
Core regulon	*ybdZ*	6.19	9.40	6.73	5.70	5.07	7.58	6.61	5.09	6.95
Core regulon	*entF*	5.43	7.89	7.96	5.38	6.35	5.63	5.78	5.62	4.64
Core regulon	*entS*	2.75	7.42	4.07	1.83	5.86	3.99	3.18	2.78	5.02
Accessory regulon	*entD*	5.47	6.68	N/A	6.12	5.29	N/A	N/A	N/A	3.02
Unique regulon	*fepE*	3.31	N/A	N/A	N/A	N/A	N/A	N/A	N/A	N/A

Note, N/A denotes not available

Supplementary Table 9 The gene expression (values in Log_2FC) of part of pan-regulon from *fur* knockout strains

**Category**	**Representative gene**	**Functional class**	***E. coli* K-12 MG1655**	***E. coli* K-12 W3110**	***E. coli* W**	***E. coli* KO11FL**	***E. coli* 042**	***E. coli* BL21(DE3)**	***E. coli* Crooks**	***E. coli* O157:H7 Sakai**	***E. coli* CFT073**
Core regulon	*fiu*	Membrane components	5.47	10.03	8.35	5.19	8.04	7.09	6.53	5.83	6.20
Core regulon	*mntH*	Transport	1.16	2.49	2.79	2.99	2.33	1.72	1.85	3.30	2.93
Core regulon	*entC*	Metabolic & biosynthetic processes	5.98	9.99	8.70	4.73	9.39	6.77	6.08	6.32	6.89
Core regulon	*entE*		5.93	9.99	9.09	4.84	7.81	5.90	6.30	6.14	6.41
Core regulon	*yjjZ*	Unknown	7.91	10.25	8.49	11.32	7.28	5.54	7.67	8.12	6.41
Accessory regulon	*ydhY*	ferredoxin-like protein	N/A	N/A	N/A	1.08	-4.68	-2.88	N/A	N/A	N/A
N/A	*oxyR*	Regulator	N/A	N/A	N/A	0.43	N/A	-0.21	N/A	N/A	N/A

Note, N/A denotes not available
